# Why multiple intelligences theory is a neuromyth

**DOI:** 10.3389/fpsyg.2023.1217288

**Published:** 2023-08-28

**Authors:** Lynn Waterhouse

**Affiliations:** The College of New Jersey, Ewing Township, NJ, United States

**Keywords:** multiple intelligences, brain, neuromyth, teaching methods, cognition, neuroscience

## Abstract

A neuromyth is a commonly accepted but unscientific claim about brain function. Many researchers have claimed Howard Gardner’s multiple intelligences (MI) theory is a neuromyth because they have seen no evidence supporting his proposal for independent brain-based intelligences for different types of cognitive abilities. Although Gardner has made claims that there are dedicated neural networks or modules for each of the intelligences, nonetheless Gardner has stated his theory could not be a neuromyth because he never claimed it was a neurological theory. This paper explains the lack of evidence to support MI theory. Most important, no researcher has directly looked for a brain basis for the intelligences. Moreover, factor studies have not shown the intelligences to be independent, and studies of MI teaching effects have not explored alternate causes for positive effects and have not been conducted by standard scientific methods. Gardner’s MI theory was not a neuromyth initially because it was based on theories of the 1980s of brain modularity for cognition, and few researchers then were concerned by the lack of validating brain studies. However, in the past 40 years neuroscience research has shown that the brain is not organized in separate modules dedicated to specific forms of cognition. Despite the lack of empirical support for Gardner’s theory, MI teaching strategies are widely used in classrooms all over the world. Crucially, belief in MI and use of MI in the classroom limit the effort to find evidence-based teaching methods. Studies of possible interventions to try to change student and teacher belief in neuromyths are currently being undertaken. Intervention results are variable: One research group found that teachers who knew more about the brain still believed education neuromyths. Teachers need to learn to detect and reject neuromyths. Widespread belief in a neuromyth does not make a theory legitimate. Theories must be based on sound empirical evidence. It is now time for MI theory to be rejected, once and for all, and for educators to turn to evidence-based teaching strategies.

## Introduction

Howard Gardner’s theory (Gardner, 1983, 1993, 2011) of multiple intelligences is widely regarded as a neuromyth (Geake, 2008; Dekker et al., 2012; Howard-Jones, 2014; Ruhaak and Cook, 2018; Blanchette Sarrasin et al., 2019; Rogers and Cheung, 2020; Craig et al., 2021; Menz et al., 2021). A neuromyth is a commonly accepted but unscientific claim about brain function that may be based on a misunderstanding of brain research findings (Dekker et al., 2012). The goal of this paper is to explain why Howard Gardner’s Multiple Intelligences (MI) theory is a neuromyth.

Specifically, critics have claimed that Gardner’s neuromyth is his proposal that humans have independent brain-based intelligences for different types of cognitive abilities: linguistic; logical-mathematical; visual-spatial; bodily-kinesthetic; musical; interpersonal; intrapersonal; and naturalistic.

Although critics have labeled MI theory as a neuromyth, little evidence has been published to support this claim. Geake (2008) noted that one neuroscience study found that the frontal lobe governed different forms of cognition. From this Geake argued the intelligences could not have separate brain networks. Howard-Jones (2014) asserted that the “general processing complexity of the brain makes it unlikely that anything resembling Multiple Intelligences theory can ever be used to describe it” (p. 818), but he did not provide evidence of the brain’s complexity. Craig et al. (2021), Dekker et al. (2012), Ruhaak and Cook (2018), and Blanchette Sarrasin et al. (2019) simply labeled MI theory as a neuromyth.

Rousseau (2021b) argued that MI theory itself was not a neuromyth. He stated that it was a “legitimate scientific theory of intelligence” (p. 4) that could be validated by finding the brain bases for the intelligences. However, Rousseau (2021b) claimed that it was a myth that matching students’ multiple intelligences to teaching methods could enhance learning.

The current criticisms of Gardner’s theory are not new (Shaler, 2006; Waterhouse, 2006), and over time Gardner has taken four distinct unsatisfactory positions to defend against criticisms of his theory. One position was to ignore criticisms. Gardner asserted “For over a decade, I was content to let MI theory take on a life of its own. I had issued an ensemble of ideas (or “memes”) to the world, and I was inclined to let those memes fend for themselves” (Gardner, 2011, p. 79). A second position was to argue that empirical support for his theory was provided by his personal reading of research: “Gardner cast a wide net that included neuroscience, cognitive science, anthropology, and evolutionary sciences. This broader view allowed Gardner to reconceptualize intelligences” (Gardner and Moran, 2006, p. 229). But personal reading of research is how theories are created, not how they are validated. Empirical support for a theory requires testing the theory. Gardner’s third position was to tell researchers to read his previous writings to find an argument for the validity of his theory (Gardner, 2020b). He stated “there is an entire 400-page book *Howard Gardner Under Fire* in which I respond to these and other critiques (Shaler, 2006). I would ask that both researchers and educators review these writings and exchanges before connecting the theory that I developed with the provocative, and contentious phrase “neuromyth” (Gardner, 2020b, p. 3).

A fourth position has been Gardner’s continuing assertion (Gardner, 2011) that MI theory could not be a neuromyth because he had never claimed there were brain bases for the intelligences. He stated “while brain evidence was cited in my original work, I have never claimed that “MI” is a neurological theory” (Gardner, 2011, p. 3). However, Gardner did make claims for the neural basis of the intelligences. Gardner argued “that the mind/brain consists of many modules/organs/intelligences, each of which operates according to its own rules in relative autonomy from the others” (Gardner, 2011, p. xxiii). Gardner also argued that patients with brain damage demonstrated that “various abilities can be destroyed, or spared, in isolation from other abilities” (Gardner, 1993, p. 7). Gardner defined each intelligence as a separate pattern of thinking, “where each operates according to its own procedures and has its own biological bases” (Gardner, 2011, p. 72). Importantly, Gardner asserted that each “intelligence …clearly involves processes that are carried out by dedicated neural networks. No doubt each of the intelligences has its characteristic neural processes” (Gardner, 2020a, p. 94). Gardner specifically argued that “MI theory demands that linguistic processing, for example, occur via a different set of neural mechanisms than does spatial or interpersonal processing” (Gardner, 2020a, p. 99), and Gardner and his colleagues (Davis et al., 2011) proposed that there should be “an atlas of the neural correlates of each of the intelligences” (p. 495). Davis et al. also predicted that “The biological basis of the theory—its neural and genetic correlates—should be clarified in the coming years” (Davis et al., 2011, p. 498).

But, to date, no neural correlates of the intelligences have been found (Waterhouse, 2006; Geake, 2008; Dekker et al., 2012; Howard-Jones, 2014; Ruhaak and Cook, 2018; Blanchette Sarrasin et al., 2019; Craig et al., 2021; Rousseau, 2021b). Consequently, until researchers find evidence that “each of the intelligences has its characteristic neural processes” (Gardner, 2020b, p. 94), MI theory is and will remain a neuromyth. And until each intelligence is shown to have its own unique brain processing, there is no basis for the superiority of MI teaching strategies. Despite the lack of evidence for multiple intelligences, Ruhaak and Cook (2018) reported that 90% of teacher trainees surveyed in the United States planned to use MI teaching strategies, and Blanchette Sarrasin et al. (2019) reported that 94% of teachers surveyed in Quebec stated they used MI theory in the classroom. Others, including Abenti (2020), Hanafin (2014), Shearer (2020a), and Yavich and Rotnitsky (2020) all have argued that use of MI theory in the classroom was effective and important. Shearer noted that many educators “continue to alter lesson plans, re-envision curriculum and design whole schools inspired by the multiple intelligences” (Shearer, 2020a, p. 49). But because MI theory has not been validated, significant efforts should be made to dispel the widespread belief in MI theory and the belief in the effectiveness of MI-inspired teaching strategies.

## Three lines of evidence undermine MI theory

Three lines of evidence presented here explain why MI theory is a neuromyth. Each line of evidence counters a specific belief about multiple intelligences. One, although there is a pervasive public belief that there are distinct multiple intelligences, studies have shown that the intelligences do not function separately from one another. Two, although many educators believe in the effectiveness of MI teaching strategies, these strategies have not been studied appropriately and cannot prove the intelligences exist. Three, although many educators and researchers believe there is a brain basis for each intelligence, this is improbable because neuroscience research has shown that the brain is organized in complex multifunction networks, and multifunction networks, as understood now, preclude the possibility of separate neural networks for the individual intelligences.

The paper begins with a brief review of the lack of standard measures for the intelligences, and then discusses each of the three lines of evidence that makes MI theory a neuromyth. Following this, the paper explains the brain’s multifunction networks. The paper concludes with a discussion of possible ways to dispel the belief that MI theory is a good basis for teaching.

## There are no standard measures of the intelligences

Having a standard measure for each of the intelligences is a requirement for exploring the validity of MI theory, and Gardner et al. (1998) did create measures to assess the intelligences, but these measures did not become standard measures. Davis et al. (2011) argued that standard measures of the multiple intelligences were needed, and encouraged the development of standard measures. Gardner, however, argued that it was up to others to create measures of the intelligences, and said he “would prefer to spend more resources helping learners understand and develop their individual intelligence profiles…. He has made the personal decision not to become directly involved in testing” (Gardner and Moran, 2006, p. 230). Gardner and Moran (2006) claimed it was hard to test the intelligences by psychometric means, i.e., “paper-and-pencil assessments” (p. 230). Nonetheless, Gardner (2011) stated that “as psychologists have increased their tools for measuring intelligence, psychometric evidence in favor of multiple intelligences has accrued” (p. 40).

Teele (1992) published the Teele Inventory of Multiple Intelligences (TIMI). This psychometric test of the intelligences was used in 1,000 schools. However, McMahon et al. (2004) evaluated the TIMI and discovered that “Reliability analyses for each of the subscales of the TIMI suggested that the instrument does not provide consistent measurement” (p. 48). They concluded that the poor reliability of the TIMI meant that it should not be used by educators.

At present there are no standard measures of the intelligences, thus individual researchers have to create their own measures for the intelligences. Unfortunately, without standard measures, MI study findings cannot be compared to one another. Also, the lack of standard measures means that no synthesis of MI research findings can be built.

## Evidence for the independence of the intelligences is lacking

Gardner (1983, 1993, 2011, 2017, 2020a) claimed that the intelligences were independent abilities, and Gardner stated that evidence for “the independence of an intelligence comes from studies that identify individuals who either excel at, or lack, a certain capacity, as well as “dedicated” neural regions that appear to subserve these capacities” (Gardner, 2011, p. 50). Gardner and Walters stated that the independence of the intelligences was a crucial component of MI theory, and they worried that if there were “a significant correlation among these faculties, as measured by appropriate assessments, the supposed independence of the faculties would be invalidated” (Gardner and Walters, 1993, p. 38).

Importantly, the independence of the intelligences is crucial for MI theory, because if the intelligences are intercorrelated they do not exist as theorized. But only a few studies have explored the independence of the intelligences, and these studies were not based on shared standard measures of the intelligences. A factor analysis by Plucker et al. (1996) did report evidence for independent linguistic, logical–mathematical and spatial subscales. However, Visser et al. (2006a) analyzed the score correlations of 200 individuals on their measures of each of the eight intelligences, and they found that test scores representing most of the intelligences were intercorrelated “despite representing different domains of Gardner’s framework” (p. 500). Visser et al. concluded that their findings “contradict Gardner’s assertion that there are at least eight independent intelligence domains” (Visser et al., 2006a, p. 501).

Gardner (2006) claimed that Visser et al. (2006a) only found intercorrelations for the intelligences because they used measures that relied on two of the multiple intelligences—linguistic and logical-mathematical. Visser et al. (2006b), however, responded that intercorrelations of the intelligences depended on the association of individual differences in task performance, and not on whether a test item used language or reasoning. Visser et al. (2006b), also noted that Gardner had not established measures for his eight intelligences, and the researchers requested that Gardner should do so. However, as noted above, no standard measures of the intelligences have yet been developed.

Almeida et al. (2010) conducted a confirmatory factor analysis on scores from a set of Gardner’s multiple intelligence assessment tasks, including linguistic, logical, visual/spatial, bodily-kinesthetic, naturalistic and musical intelligences, and scores from the Battery of General and Differential Aptitudes (BADyG), including reasoning, memory, verbal aptitude, numerical aptitude and spatial aptitude, in a sample of 294 children. Almeida et al. (2010) found that there was no general common factor across the two test batteries. Instead, the researchers found that there were two factors, one for the BADyG measures, and another for the multiple intelligence assessment scores. The single multiple intelligence factor suggests that the individual intelligences are not independent of one another.

Castejon et al. (2010) explored the construct validity of the multiple intelligences through confirmatory factor analysis of measures of the intelligences that they had developed. They reported that no model exhibited a totally satisfactory fit to the empirical data, but the best fitting factor structure was a model that was based in the intercorrelation of the intelligences, along with some individuation. The researchers (Castejon et al., 2010) concluded that their analyses demonstrated that the intelligences are not truly independent of one another. Consequently, to date, no clear division of cognition into separate intelligences has been proven.

## Belief in MI teaching strategies is strong but is not supported by valid evidence

Educators have continued to use MI teaching methods because they believe that there are separate brain-based intelligences that vary in strength for each individual (Attwood, 2020; Mavrelos and Daradoumis, 2020). These beliefs support the idea that teaching to a student’s stronger intelligences will be more effective. For example, Shearer stated that “multiple intelligences serve as ‘levers’ to personalize important cognitive processes underlying learning” (Shearer, 2020a, p. 57), and Gardner argued that the “intelligences can function…as the preferred means for inculcating diverse subject matter” (Gardner, 2011, p. 409). He asserted that computer programming would be better taught to an individual’s strongest intelligence. He stated that “an individual with a strong musical bent might best be introduced to programming by attempting to program a simple musical piece” and that “An individual with strong spatial abilities might be initiated through … a flow chart or some other spatial diagram” (Gardner, 2011, p. 409). A ‘strong musical bent’ and ‘strong spatial abilities’ mean better musical and spatial intelligence skills which stem from more effective neural networks dedicated to musical intelligence and visual-spatial intelligence.

Even though Gardner and colleagues stated that “Individualized education does not depend on the existence of MI theory” (Davis et al., 2011, p. 499), teacher training continues to promote MI theory as an effective basis for individualized lessons (Sheahan et al., 2015; Armstrong, 2018; Abenti, 2020; Attwood, 2020). Myriad educators and researchers believe that “teachers should be encouraged to learn about student engagement strategies which MI theory provides” (Attwood, 2020, p. 6), and they have argued that individualized and non-individualized MI teaching strategies are more effective than standard instruction. For example, Yavich and Rotnitsky (2020) asserted that MI teaching strategies can significantly contribute to “the development of logical, critical and creative thinking abilities, as well as the development of high levels of thinking” (p. 111), and Abenti (2020) reviewed studies of MI teaching strategies and concluded that MI teaching methods are the best way to “educate the most people in any education environment” (p. 32).

## Problems for studies of MI teaching strategies

There are many published papers addressing the use of MI theory in the classroom. A search of databases on January 14, 2023 using the query ‘multiple intelligences classroom’ for the time period 1983 to 2022 found 407 papers on Education Full Text, and 92 papers on PubMed. Most of the published classroom studies found improved learning through use of MI teaching methods. Bas (2016), Batdi (2017), and Ferrero et al. (2021) conducted meta-analyses of studies of the effectiveness of using MI in the classroom, and all three meta-analyses reported many studies with positive results for MI teaching. Some MI teaching strategies have focused on teaching students the different intelligences. For example, Winarti et al. (2019) compared student improvement in the eight intelligences when taught by MI strategies with student improvement when not taught using MI strategies. Students taught by MI strategies showed significant improvement in measures of six intelligences—intrapersonal, interpersonal, kinesthetic, visual spatial, musical intelligence, and linguistic intelligence. By contrast, there was no improvement in the control group for any of the eight intelligences.

Most MI teaching strategies, however, follow Gardner’s (2011) argument that the intelligences be used “for inculcating diverse subject matter” (p. 409). For example, Sheahan et al. (2015) conducted a study of first year nursing students who were learning clinical skills. The researchers reported that 46 first year nursing students taught by multiple intelligence strategies received significantly higher scores on three structured clinical examination tests than a control group of 44 first year nursing students taught by conventional methods.

However, the apparent success of matching a student’s stronger intelligence to specific task can cannot prove the existence of multiple intelligences because there is no evidence that the intelligences are independent (Visser et al., 2006a; Almeida et al., 2010; Castejon et al., 2010), and no evidence that there is a brain basis for each intelligence (Waterhouse, 2006; Geake, 2008; Dekker et al., 2012; Howard-Jones, 2014; Ruhaak and Cook, 2018; Blanchette Sarrasin et al., 2019; Craig et al., 2021; Rousseau, 2021b). Davis et al. argued “because any educational intervention is multifaceted, it is not possible to attribute school success or failure strictly to MI interventions” (Davis et al., 2011, p. 498). In fact, it is unlikely that *any* enhanced learning is due to the existence of multiple intelligences. Where MI intervention studies have reported evidence of enhanced learning, it is likely that learning is actually enhanced by other well-known methods that have been tested and found effective. These include, but are not limited to, the extended repetition of information, greater student attention to novel teaching strategies, increased personal attention from the instructor, and the greater enthusiasm of teachers using new teaching methods. Importantly, cognitive neuroscience research has established that repetition enhances learning (Zhan et al., 2018; Adams and Delany, 2023), and that there is a novelty ‘switch’ in the brain that enhances the processing of something new in the environment (Gómez-Ocádiz et al., 2022). Learning is also enhanced when there is individual attention to a student by a teacher (Schacter, 2000), and when excitement occurs at the time the material is presented (Perugini et al., 2012; Leventon et al., 2018).

Studies of MI teaching strategies (Ferrero et al., 2021) have not controlled for any of these many likely alternate causes for enhanced learning. Also problematic, Ferrero et al. (2021) found that most of the MI teaching studies they reviewed in their meta-analysis could not prove the effectiveness of MI for enhancing learning because these studies did not have large enough sample sizes, appropriate control groups, or reliable and valid outcome measures. The researchers warned that sound studies of MI in the classroom were needed before “its use in the classroom can be recommended or promoted” (Ferrero et al., 2021, p. 12).

## There are no empirical studies of the brain basis of MI theory

As stated above, Gardner’s claims for the neural basis of the intelligences led educators and researchers to believe that each of the eight intelligences had a distinct brain basis. Armstrong (2018) stated the intelligences were “eight relatively autonomous brain systems” (p. 14), where each intelligence has “its roots deeply embedded in the evolution of human beings” (p. 22) and Shearer (2018) claimed that “each of the eight intelligences have unique neural architectures” (p. 3). However, Rousseau (2021b) and Waterhouse (2006) argued that empirical research was needed to test the belief that there is a dedicated brain network for each intelligence. Ideally, a brain validity test of MI theory should measure brain processing while individuals are engaged in behaviors that characterize a particular intelligence (Waterhouse, 2006).

Although Davis et al. (2011) had declared that the brain basis of MI theory “should be clarified in the coming years” (p. 498), and although Rousseau (2021b) argued that studies should test for brain bases for the multiple intelligences, Gardner (2020b) made two claims that, if accepted, would effectively block future studies to test MI theory.

One, he claimed MI theory cannot be evaluated by research findings from cognitive psychology and neuroscience. Even though Gardner and Moran (2006) declared that MI theory “encompasses cognitive and developmental psychology, differential psychology, (and) neuroscience” (p. 227), nonetheless, Gardner (2020b) argued that MI theory could not be evaluated by research findings from cognitive psychology and neuroscience. He claimed this research used study samples that were too risky. He asserted that “Much neural evidence comes from studies of animals…. But even when the population that has been studied consists of *Homo sapiens*, generalizations are risky” (Gardner, 2020b, p. 3). However, Gardner’s concern for the riskiness of generalizations from these study samples is unfounded. Research using samples of animals and humans have been the source of most of our current valid knowledge of the brain basis of cognition (Gazzaniga et al., 2018). Thus, MI theory *can* be evaluated by findings from cognitive psychology and cognitive neuroscience research. If his theory cannot be evaluated by scientific findings it remains a personal philosophy and an untested speculation.

Two, Gardner claimed that because MI theory is a scientific theory with educational implications, it cannot be tested for validity by standard research methods. Gardner (2020b) argued that a scientific theory with educational implications, such as MI theory, was different from a purely scientific theory. Gardner asserted that purely scientific theories could be supported or countered by research, but theories about the brain with educational implications could not be similarly tested because an “educational claim turns out to be either circular or vacuous. Indeed, once one moves from “science” to “education” one has indubitably entered the realm of cultural values” (Gardner, 2020b, p. 2).

However, a scientific claim about the brain with educational implications *can* be supported or countered by research without entering the realm of cultural values. For example, one important scientific theory about the brain that has educational implications argues that student repetition of information enhances learning through brain activity changes. For example, a study by Zhan et al. (2018) found that repetition of information did improve long term learning, and repetition was correlated with significantly increasing activation in the hippocampus, as well as increased connectivity between the hippocampus and other brain regions. Another important scientific theory about brain activity with educational implications is that learning is enhanced by brain activity that occurs while we sleep—even during naps. Klinzing et al. (2019) reviewed a wide range of studies of the effect of sleep on learning, and concluded that what we learn while awake is repeated during slow wave sleep (SWS) and this neuronal replay while we sleep strengthens the information flow to brain networks, thus enhancing learning. Importantly, studies like those of Zhan et al. (2018) and Klinzing et al. (2019) can be supported or countered by future research.

Although future studies to explore the brain basis of the intelligences could be conducted, they will not stem from past or ongoing research, because, as noted above, there is no evidence that empirical brain validity studies have been done (Waterhouse, 2006; Rousseau, 2021b). While it is possible there may be MI neural validity studies, none have been cited by Gardner (1993, 2011, 2020a, b). Moreover, evidence that validity studies have not been done can be seen in the lack of published studies. On January 10, 2023, using the query “validity Gardner multiple intelligences,” for the time period 1983–2022, the database PubMed yielded only two published papers, neither of which were validity studies. And for the same query on the same date, the database Education Full Text yielded just nine papers published between 1983 and 2022, and among these nine papers, only one, Shearer (2020b), provided an assessment of MI neural validity. However, Shearer’s paper (Shearer, 2020b) does cite another Shearer study—Shearer and Karanian (2017).

Shearer and Karanian (2017) and Shearer (2020b) reported that characteristics of the intelligences were aligned with the functions of cognitive brain networks. However, because alignment studies do not measure brain processing while individuals are engaged in activities that characterize a particular intelligence, these studies are not true validity studies, and thus cannot prove that the intelligences have dedicated neural networks.

Shearer and Karanian (2017) reviewed 318 studies of brain regions and reported that brain region functions were aligned with the proposed functions of each of the eight intelligences. They concluded that their analysis of the 318 studies provided “robust evidence that each of the eight intelligences possesses its own unique neural architecture” (Shearer and Karanian, 2017, p. 221). However, there are problems with Shearer and Karanian’s methods and conclusions. First, the researchers did not adequately describe how they selected the 318 neuroscience studies. They simply stated “Several extensive meta-analysis and topic reviews served as guides for finding pertinent studies in the target area” (Shearer and Karanian, 2017, p. 212). Second, the researchers claimed the unique neural architecture for linguistic intelligence included eight brain regions—temporal cortex, frontal cortex, parietal cortex, occipital cortex, the subcortical region, the cerebellum, the cingulate cortex, and the insular cortex. These eight regions comprise nearly the entire brain. Although language processing involves many links between multifunction networks across different regions of the brain (Popham et al., 2021; Rolls et al., 2022; Wang et al., 2022), the entire brain is not a unique neural architecture dedicated to language processing.

A similar alignment study was conducted by Shearer (2020b). He reviewed 48 studies of brain connectivity, and argued that “neural regions cited by Gardner’s original research in 1983 are among the same brain structures identified by modern neuroimaging technologies for each intelligence” (p. 142). He stated that “7–15 neural networks were found to be well aligned with seven of the eight multiple intelligences” (Shearer, 2020b, p. 127). Shearer (2020b) claimed that these seven alignments were: (1) visual–spatial intelligence with visual networks and subnetworks; (2) kinesthetic intelligence with primary motor, sensorimotor, cerebellum, and basal ganglia networks; (3) logical-mathematical intelligence with fronto-parietal and executive control networks; (4) musical intelligence with the auditory network; (5 and 6) intrapersonal and interpersonal intelligences with a network for internal self-awareness and social perceptions; and (7) linguistic intelligence with the language network.

The key problem with both Shearer and Karanian’s study (Shearer and Karanian, 2017) and Shearer’s study (Shearer, 2020b) is that the brain networks they have described are significantly oversimplified. Gardner’s linguistic intelligence cannot be aligned with “the language network,” because there is no isolated single “language network” in the brain (Popham et al., 2021; Rolls et al., 2022; Wang et al., 2022). Networks for language and other forms of cognition are shared, and language processing occurs in the same networks that also process many other cognitive tasks such as mathematics, music, and logic, and varied perceptual and motor skills as well. For example, syntax, or word order, is generated by brain network activity in the inferior frontal gyrus region and the left orbitofrontal region. And syntax processing networks are driven by two word-level networks: the network that governs visual object meaning; and the network that governs visual motion, and auditory, and somatosensory semantic processes (Rolls et al., 2022). Adding to the complexity of these processes, the brain networks governing syntax also serve as hubs for social perception and cognition, including the perception of faces and human motion, as well as the understanding others’ actions, and mental states.

Another example of the complexity of language processing in the brain is that the network for understanding the meaning of verbs also provides control for our motor actions. Specifically, Wang et al. (2022) reported that the inferior parietal lobe supports verb generation and verb retrieval, and the inferior parietal lobe also processes abstract information of object-directed actions, and it governs action organization and understanding the intention of others’ actions.

Because the varied neural networks that govern language processing also govern many other forms of cognition, a unitary dedicated language network cannot be isolated.

## Multifunction brain networks

There are complex multifunction networks for all aspects of cognition. However, this complexity was not evident 40 years ago. Neuroscientists in the 1980s and 1990s theorized that there were modular cognitive mechanisms in the brain. Fodor (1983) defined cognitive modules as “domain-specific, innately specified, hardwired, autonomous” (p. 36). Fodor (1983) also defined cognitive modules as “informationally encapsulated” (p. 64) containing specific information within a “fixed neural architecture” (p. 98), and Sperber (1994) similarly stated that “The function of a module is to process a specific range of information in a specific manner” (p. 52).

Thus, Gardner’s MI theory was not a neuromyth in 1983 or 1993 because it was based on then current theories of brain modularity for cognition, and at that time few researchers were concerned by the absence of neural validity studies (Waterhouse, 2006). However, since then evidence has demonstrated that the brain is not organized in cognitive modules uniquely dedicated to different forms of content (Anderson, 2014; Sporns and Betzel, 2016; Zerilli, 2017; Elimari and Lafargue, 2020; Holyoak and Monti, 2021; Wang et al., 2021; Roy et al., 2022). Therefore, the lack of brain validity for the multiple intelligences has become a serious concern, and has led to the claims that MI theory is a neuromyth.

The word module is still used in cognitive neuroscience research. However, its current use does not mean a cognitive module as proposed by Sperber (1994) or Fodor (1983). Instead, the word module now is applied to many varied groups of biological elements within the brain, including groups of interacting proteins, or sets of genes that work together in cellular networks, or neuron assemblies that are “building blocks in the organization of brain networks” (Sporns and Betzel, 2016).

The idea of complex networks for cognition was proposed by Lieberman (2002). He suggested that assemblies of subcortical neurons in basal ganglia and cerebellum, and in many separate cortical regions shared control of many different complex behaviors including walking, talking, gesturing, reasoning, speaking, tool-making, and comprehending the meaning of sentences. As discussed above, there are now many lines of evidence demonstrating the shared complex brain networks for the processing of information. Wang et al. (2021) reported evidence for the current model of switching between brain integration and segregation. This model argues that individual forms of cognition occur in a process of constant shifts between large networks and small networks, and not in separate content-dedicated static modules. Roy et al. (2022) found evidence that a single memory is stored in multiple coded information units that are widely distributed across the brain. Holyoak and Monti (2021) reported evidence that mental representations of ideas, regardless of specific content—i.e., language, logic, and mathematics—involve neural activity that is distributed across the parietal and frontal lobes of the brain. Anderson et al. (2013) conducted a meta-analysis of more than 2,000 functional imaging studies of the brain and discovered functional diversity at every location in the brain. Specifically, the researchers reported that language, vision, emotion, and mathematics did not have discrete local specialization. Instead, they found that each region of the brain supported a varied array of tasks, where multiple regions and networks supported different components of cognitive, perceptual, and motor tasks or skills.

A likely cause for the functional diversity of many brain networks is the evolution of the brain through exaptation. Exaptation is the reuse of neurons in existing brain networks as the basis for new networks to support the additional processing activity needed for new skills and new knowledge (Zerilli, 2017). Exaptation is adaptive because it reduces the amount of glucose energy needed to build new brain networks. It is likely that exaptation is the cause of the layering of varied perceptual, cognitive and motor functions in multi-use brain networks that govern many varied forms of thinking and action. And Elimari and Lafargue (2020) claimed that the most recently evolved cognitive skills, such as language and mathematics, rely on a “greater number and diversity of neural structures” (p. 11).

## What will convince educators that MI theory is a neuromyth?

Even though Gardner has consistently argued that the intelligences are real (Gardner, 1983, 1993, 2011), surprisingly Gardner (2011) also asserted the “intelligences are fictions—at most, useful fictions—for discussing processes and abilities that (like all of life) are continuous with one another” (p. 74).

As outlined here, studies have not confirmed that the intelligences are real. Factor studies have not shown the intelligences to be independent. Studies of MI teaching effects have not explored alternate causes for positive effects and have not been conducted by standard scientific methods. No research has directly looked for a brain basis for the intelligences. Most important, neuroscience research has shown that the brain is not organized in separate modules dedicated to specific forms of cognition. Consequently, MI theory is a neuromyth that should not be taught to teachers, and MI teaching strategies should not be used in the classroom.

Because so many educators use and value MI teaching strategies, it is likely that many teachers and researchers do not know the evidence that has made MI theory a neuromyth. Craig et al. (2021) reported that only 25% of teachers surveyed in Canada, the United States, and the United Kingdom believed MI theory was a neuromyth. Dekker et al. (2012) found that teachers who knew more about the brain still believed education neuromyths. The researchers also reported that “teachers who are enthusiastic about the possible application of neuroscience findings in the classroom, often find it challenging to distinguish pseudoscience from scientific facts” (Dekker et al., 2012, p. 6). And Ruhaak and Cook (2018) found that teachers’ “knowledge of neuromyths—not general knowledge of the brain—was associated with greater likelihood of implementing more effective instructional practices than ineffective, neuromyth-based approaches” (p. 160).

Many interventions have attempted to change teachers’ belief in neuromyths. Rousseau (2021a) conducted a comprehensive review of interventions for fostering conceptual change in teachers that would improve their ability to recognize and refute neuromyths. Rousseau (2021a) reported that “The high prevalence of beliefs in neuromyths among educators did not decline over the past decade” (p. 9), and his review of intervention research led him to conclude that much more data was needed to understand what teaching processes would be most effective in changing teachers’ minds. Rousseau’s (2021a) review of interventions revealed that neither the direct refutation of neuromyths nor training in neuroscience had been shown to be consistently effective in reducing students’ belief in neuromyths. Rousseau reported, however, that although an undergraduate course in neuroscience was “insufficient to reduce beliefs in neuromyths” (Rousseau, 2021a, p. 8), “training in the context of teacher professional development (workshops, seminars) looks promising” (Rousseau, 2021a, p. 8). Dubinsky et al. (2022) argued that teachers needed to learn current concepts of neuroscience, and posited that “where teachers are given the opportunity to discuss what the physiological neuroscience concepts might mean for their own classrooms, strong connections between neuroscience principles and effective pedagogical practices have been documented” (p. 272). Rousseau (2021a) stated that “Given that intuitive/anecdotal evidence might contribute to consolidate false beliefs through a variety of cognitive biases (availability, familiarity, confirmation biases), some intervention approaches focus upon making educators more aware of the propensity of the human mind to rely on intuitive thinking at the expense of rational thinking” (p. 6). Rousseau (2021a) argued that it is important to break through the intuitive beliefs many students have for neuromyths, and he recommended that teacher training programs address anecdotal evidence and focus on cognitive biases.

Craig et al. (2021), Dekker et al. (2012), Goswami (2006), Grospietsch and Mayer (2018), Howard-Jones (2014), Ruhaak and Cook (2018), and Blanchette Sarrasin et al. (2019) have also outlined various possible strategies to undo or reduce belief in neuromyths. Howard-Jones (2014) argued that that neuroscientists and educators should work together to establish a new field of educational neuroscience which could address neuromyths and “encourage scientific insight regarding the relationship of neural processes to the complex behaviors that are observed in the classroom” (p. 822). Dekker et al. (2012) recommended that research should discover what sources, such as books, colleagues, and commercial education companies, lead teachers to know and believe neuromyths. Dekker et al. (2012) also recommended that there be interdisciplinary connections between neuroscientists and educators. Goswami argued that learning current neuroscience findings was very important for educators (Goswami, 2019), but had earlier cautioned that neuroscientists were not appropriate instructors for dispelling neuromyths because they provide “too much data” (Goswami, 2006, p. 412), and “are not necessarily gifted at communicating with society at large” (p. 6). However, Goswami (2006) recommended that educators should establish a group of “ex-scientists with an interest in education, perhaps attached to universities or to national education departments. They could fulfill a dual role: interpreting neuroscience from the perspective of and in the language of educators, and feeding back research questions and ideas from educators to neuroscientists” (p. 413).

Goswami (2006) cautioned that “Most teachers prefer broad brush messages with a ‘big picture,’ and being ‘told what works’” (p. 412). Blanchette Sarrasin et al. (2019) recommended that teachers should be trained in critical thinking, and that education textbooks should include current knowledge about the neuroscience of learning, and clear refutations of neuromyths. Similarly, Craig et al. (2021) argued that teacher training should include information that helps teachers be critical thinkers about educational neuroscience. And Craig et al. (2021) asserted that because school psychologists are trained in evaluating research and determining the evidence base for interventions, therefore “school psychologists are obligated to take action to de-implement harmful or wasteful practices in schools, drawing on their diverse and multi-faceted training across the domains of education, psychology, and neuroscience” (p. 136).

Dekker et al. (2012) proposed that it was important to develop and test interventions that explain neuromyths and teach educators evidence-based teaching methods, and this is happening with varying degrees of success (Rousseau, 2021a). Educators need to learn to detect and reject neuromyths, and neuroscientists and educators should work together to establish a new field of educational neuroscience. Ideally, Gardner or an MI theory adherent should publish a paper or book in which the lack of evidence for multiple intelligences is accepted, reviewed and addressed. However, this is unlikely to happen. Nonetheless, the widespread belief in multiple intelligences inhibits the development of effective, evidence-based teaching strategies, and must be dispelled.

## Conflict of interest

The author declares that the research was conducted in the absence of any commercial or financial relationships that could be construed as a potential conflict of interest.

## Publisher’s note

All claims expressed in this article are solely those of the authors and do not necessarily represent those of their affiliated organizations, or those of the publisher, the editors and the reviewers. Any product that may be evaluated in this article, or claim that may be made by its manufacturer, is not guaranteed or endorsed by the publisher.

## Author contributions

The author confirms being the sole contributor of this work and has approved it for publication.
